# Medicine Men of the World

**Published:** 1887-11-26

**Authors:** 


### Medicine Men of the World.

By the Man in the Crowd.

### IV.—SAVAGE AND CIVILISED PRACTITIONERS.

(*Continued from p.* 111.)

In a previous article reference was made to points of correspondence between the medical practice of savage and civilised. A very striking illustration of this will, no doubt, have occurred to many readers familiar with Dr. Livingstone's great book on his African missions. The diseases known to the Bakwains, says the famous explorer, are remarkably few. Among those few, however, are small-pox and measles, which, about twenty years previous to Livingstone's visit to them, had passed through the country, and committed great ravages. As a specific against small-pox, the natives, he found, employed inoculation in the forehead with some animal deposit; in some parts they employed the matter of the small-pox itself. Where they got the idea from, Livingstone said he could not conceive, as it was practised at a time when they had neither direct nor indirect intercourse with the missionaries, who seemed to be the only people through whom any rumours of European medical practice could have reached them. That some faint echo of discoveries creating great stir in civilised countries had in some mysterious way reached them, seems, however, more probable than that native medicine-men should have conceived the idea for themselves; and the poor Bakwains afford one more illustration of Pope's immortal though much-abused aphorism, "A little learning is a dangerous thing." In one village, says the narrator, they seemed to have selected a virulent case for the matter used in the operation, for nearly all the village was swept off by the disease in a malignant confluent form.

The medicine-men of the Bakwains seem to be among the most respectable of practitioners. They inherit their profession as an heirloom from their fathers and grandfathers, says Livingstone, and they generally possess some valuable knowledge, the result of long and close observation. This, in truth, is the case with medical practitioners very generally throughout the uncivilised world. It is only here and there that the profession is hereditary, but wherever medicine has attained to the dignity of a cult, it is generally based on the possession of actual knowledge acquired by close observation, and though very commonly mixed up with debasing superstition and the grossest imposture, usually possesses at least enough of real medical skill to sustain its influence. That at the back of much imposture and meaningless ceremonial, calculated only to inspire superstitious dread, there is often an acquaintance with many important secrets of nature, both in the human frame and in the natural world around, there are many facts to prove. Very generally, of course, it is a knowledge of the vegetable world which serves the medicine-men, and, as a rule, in any case of disease it is not until he has tried the potency of all the roots and herbs in his pharmacopœia that he has recourse to the more mysterious hocus-pocus of his profession. The knowledge of the medicinal properties of plants possessed by the doctors of savages is often very extensive, and such as to enable them to produce effects which for good and evil are well calculated to secure for them the utmost awe of their patients. A highly credible witness, residing on the border-lands of the Indians, relates some very singular results of drugs employed by the medical practitioners of some of the native tribes. On one occasion he had a young Indian girl in his kitchen for some years. Early in her service so many of her people came to visit her that she was imperatively ordered to close the door against all visitors whoever they might be. It so happened that the first visitor after the ukase had gone forth was an old medicine-man, who was deeply affronted by finding himself excluded, and who went away threatening the direst revenge. Nothing was heard of him for some months, when he turned up again, apparently quite oblivious of the insult he had complained of, and cordially shook the girl by the hand. She happened at that time to have a slight wound upon the hand seized and squeezed within his own, and his pressure occasioned her some pain. On disengaging herself from him, the girl observed with dismay that in the palm of the hand with which he had grasped her own there was a black patch, and she instantly suspected that he had poisoned her wound. Having attained his object, the savage frankly admitted that her suspicions were well-founded, and he left her with the assurance that she would break out in black blotches for one month every year forever afterwards. Twelve months from the date of that meeting the predicted blotches appeared, and they continued to disfigure the body of the girl for a month, as foretold, and then, without having occasioned much pain, they disappeared. This manifestation of poisoning is said to have occurred annually so long as the girl continued under the observation of the narrator.

In another case, an old man declared himself to have been the victim of an act of revenge, which, if his belief may be accepted as well-founded, may possibly explain the phenomenon of a party of hairy people exhibited not long ago in London. He also had offended a medicine-man, who had administered a decoction of certain herbs, which had had the effect of producing hair all over his body—a thick coating, nearly an inch long, over every part of his person except about an inch round the eyes. What a noble savage that old medicine-man would have been! How he would have been fêted and fed and entertained by learned societies, if he could have come over here with his decoction of herbs, and applied it locally to our bald heads! With what breathless interest his first experiments on the adventurous few would have been watched, and what a glorious sensation it would have been for society if the wicked old man who succeeded in producing the thick coat of hair had failed to confine the action of his decoction to the crown of the head, and had wrapped up a few Society swells in the hair surtouts of so many ourang-outangs!

Another very curious effect of medicine compounded by savage practitioners is said to have been a sort of paralysis of the muscles of the face. Medicine was given to a woman without her knowledge, and with the distinct object of producing this specific effect, and without any reference to disease; and, according to Mr. H. M. Robinson, a Government officer at Winnipeg, she soon became as expressionless as a mask—"only the eyes moved, and as they were intensely black and rather sparkling, the ghastly deformity was rendered the more glaring. The most singular effect, however, was produced by her laugh. She was a jolly, good-natured squaw, and laughed upon the slightest provocation. Her eyes sparkled, and her 'Ha! ha!' was musical to a degree; but not a muscle moved to denote the merriment on that expressionless face. One felt that someone else laughed behind that rigid integument. No idea could be formed of what she thought at any time." What splendid possibilities are suggested by this barbarian drug! Only think what an invaluable face for a diplomatic agent might be provided by means of it! How many political treaties that never were concluded might have been successfully carried through, how many political plots might have been formed or frustrated, how many state secrets that have been proclaimed upon the housetops might for ever have remained unrevealed to history, if only we could always have kept their countenances as this Indian squaw was enabled to do! A single bottle of this drug would be a great power in the world of diplomacy and statecraft.

(*To be continued.*)

